# Young people’s perspective on the influence of alcohol, tobacco, vaping and fast food industries

**DOI:** 10.1016/j.fhj.2025.100256

**Published:** 2025-06-30

**Authors:** Joseph Dickenson, Sean Devlin

**Affiliations:** Wimbledon College, London, England

**Keywords:** Socio-Economic Determinants of Health, Commercial determinants of health, Life Experiences, Ultra-Processed Foods, Vapes

## Abstract

As young people, alcohol, tobacco, vapes and fast food are everywhere in our lives. They are commonplace at social events, in our media, and in adverts; explained away as part of growing up. Many young people are unaware of the health risks of these substances, partly due to the success of industry marketing. For example, most people are aware of the risks to their livers from drinking alcohol, but far fewer of us realise the small scale of consumption required.

The rise of vaping and fast food in particular has been accelerated by their low cost and widespread availability. As teenagers, most of us have limited spending power – our income comes from Saturday jobs or a handful of hours of shift work, so processed foods are the easiest choice. Vapes are easily available on most high streets, and young people often pass shops that sell them on their way to school. Society’s casual attitude towards these promotes more consumption and obscures the risks. Our social media feeds influence us all, in a myriad of ways, ranging from athletes promoting an ideal body image, to companies sponsoring adverts for their products, to influencers casually showing alcohol, smoking and vaping as part of their regular lives.

We explore solutions such as tighter regulations on packaging; clearer labelling of the risks of substances can help young people to make healthier, more informed decisions. We propose that vapes be subject to the same regulations as tobacco on storage, and mandating drab packaging; moving away from the bright, colourful designs that seem designed to target children specifically. Increasing the duty paid on these products could be beneficial, but it risks pushing consumers to potentially more risky alternatives, and further widens the socio-economic divide between rich and poor. We also suggest that teaching children to think critically from a young age will enable them to make better decisions about their health, as well as many other aspects of their lives.

As young people, alcohol, cigarettes, and vapes are everywhere in our lives. They are ubiquitous at social events, in movies and on billboard adverts. Living in a country with one of the worst rates of underage drinking in the world, drinking is portrayed as a fact of life, something that everyone does. Harm-reduction initiatives for young people focus on resisting peer pressure and responsible consumption, upholding the view that everyone will be drinking. Smoking, while less common than it used to be, still enjoys an air of rebellious glamour. It is gradually being replaced by vaping, with its bright colours and wide variety of flavours, drawing in a new generation. All three have become status symbols, synonymous with being more grown-up and entering adulthood.

Part of this is down to clever marketing. As consumers, we have been convinced that the weight of responsibility lies on our shoulders, and we’re encouraged to ‘enjoy responsibly’ (emphasis on ‘enjoy’). Companies have succeeded in making the long list of risks associated with their products. For example, alcohol is a risk factor for liver damage, brain damage, cancer and so much more. However, young people are given the impression that the risks of drinking are largely down to the impaired decision-making of those under the influence; – drink-driving, unprotected sex, drunken aggression, and so on. As over-confident teenagers, we rationalise many of these away; ‘so many people smoke and don’t get cancer’, ‘just a few won’t hurt’, and, ‘I can stop whenever I want’. We are of course aware that alcohol can damage our livers, but very few realise the small scale required to do irreversible harm to our bodies. We don’t need to be consuming vast quantities on a regular basis to cause noticeable damage.

Perhaps the scale of the industry influence is more obvious to the older generations, who have more experience of the world. Or perhaps not – after all, plenty of our parents and relatives smoke, drink or gamble too, and their attitudes influence ours. Nonetheless, very few of our peers were aware of the scope of influence of these industries when we spoke with them. For us, the Dangerous Liaisons discussion that we attended about the commercial determinants of health represented an abrupt awakening to the realities of industry influence in our lives. Travelling back together, and in the following days, we were acutely aware of the little ways in which we were being influenced. Apps from companies such as McDonald’s on our phones, offering us exclusive deals if only we would come back before the week was out. Vouchers tagged on the end of KFC receipts, promising a cheaper meal on our return. Corner shops, phone repair shops, even the stationery shop in the shopping centre, all now advertising vapes in their windows.

On a weekend getaway with friends, we used a luggage store that operated out of a vape shop, with floor-to-ceiling shelves of all kinds of salts, pods and cartridges. We were surprised to find a queue of customers from all backgrounds, and the sales assistant fielding all kinds of questions, revealing an element of the discerning vape connoisseur. Vaping has shifted from being a way of quitting smoking to a fun, social activity. Speaking to our peers, we uncovered a range of reasons for vaping: use as a stress reliever, for those struggling to cope with exam pressure; as a weaker and more socially acceptable laxative to replace purging (taking laxatives or inducing vomiting, to facilitate weight loss) for someone struggling with an eating disorder. For many, it’s part of a fun night out – where the previous generation smoked and drank on their Friday nights, we vape and drink instead. Others realise that they are stuck in a cycle, no longer doing it for fun and are actively trying to reduce their consumption. Some of them picked up on it from a very young age, copying parents who had left their vape out, not realising that the flavour they enjoyed was nicotine. Most of those that we spoke to source their vapes from legitimate shops, by virtue of being 18 themselves, posing as someone who is, or getting a friend to buy it for them. One even received them from a parent, hinting at the widespread lack of awareness of potential risks. Tighter regulations and better information at the point of purchase might help address these issues.

Part of the accessibility of vapes is down to their cost, particularly compared to alcohol and cigarettes, as they are not taxed in the same way. As most of us are in full-time education, our only income is through pocket money, or a few hours of shift work slotted around the school week. Learning about taxes in school, some lamented the tobacco duty’s impact on their wallet – pointing to the efficacy of higher tax on cigarettes and alcohol in reducing their consumption, especially in young people. However, vapes are not subject to the same taxes and as such are much more appealing to our generation – something that came up several times when asking people why they vape rather than smoke. Unfortunately, those caught up in addiction are likely to pay regardless of the price, contributing to a vicious cycle.

Similarly, the price of ultra-processed or fast food is often far below that of their unprocessed counterparts. Quick, cheap and easy, they are the go-to for many of us. Fast food shops are commonplace, especially so in less affluent areas, and their doors are open at all hours. It creates a sense of familiarity and becomes normalised, in some cases becoming a tradition – many families have a takeaway on a Friday night. In talking to our peers, some spoke of the vendors like extended family, helping them out in a tough time. However, the over-consumption of these products is a result of wealth inequality, and they become a temporary solution to a permanent problem, funnelling money away from poorer areas to shareholders of large corporations. Young people struggle to opt for healthier alternatives, leading to a knock-on effect in their development and making it harder to escape poverty. Open late, takeaways are often popular choices for people on a night out, and there are few alternatives available. Similarly, ultra-processed ready meals are often the first choice for families on a budget, or who struggle to find the time to cook, or who don’t have the skills or knowledge required to cook a meal from scratch. Many people are aware that these aren’t good for us, but the lack of readily available, similarly priced, healthy alternatives forces our hand. This contributes to a rise in excess body weight and obesity, with a knock-on effect on our healthcare systems.

Such excess body weight is often labelled as a personal failing, resulting from a lack of discipline. Across social media, fitness and lifestyle influencers declare that all that is needed to lose weight is to ‘eat less and move more’, and to maintain a calorie deficit. This often-repeated phrase is enticing in its simplicity, driving us to engage and follow these creators, pushing their content out to a wider audience. Taken at face value, athletes and personal trainers promoting exercise and healthy eating is a cause for celebration. But packaged up with the workout routines and meal plans is the implication that this is what the average body looks like. Growing up with the internet, we know not to believe everything we see online, we know that people edit their photos, and we know that they hide their use of performance-enhancing drugs. Yet we are still under the impression that the bodies we see in our mirrors should match up to the bodies we see on our screens. Under the microscope of comparison, we obsess over our insecurities, and four in 10 teenagers worry about their body image as a direct result of social media^1^ – something that was reflected in talking to our peers.

This contributes to a rise in disordered eating, from obsessions over precise quantities of macronutrients, to calorie counting, to purging. Through speaking to friends, we have become aware of the glamorisation of eating disorders. Likely coming from a place of their own insecurities about body image, their peers or family around them often praise their self-control, and express their desire to be as ‘good’ as them, once again feeding into the myth of weight being directly representative of an individual’s self-control. But there is another side to it too – social media self-help advice advocates caffeine and nicotine, through smoking or vaping, as methods for speeding up metabolism and thereby reducing excess body weight.

Our screens influence us in many ways – smoking, drinking and vaping feature heavily in the media that we consume. Coming-of-age movies often feature the rebellious teen, smoking or drinking at a party. As young people, we are searching for a sense of belonging, finding the people that we ‘click’ with, and we are drawn into the association with socialising, of wanting to be part of the popular ‘in’ crowd. Smoking is falling; we have been put off by the drab packaging, graphic images, and seeing the impact that it’s had on our older relatives. But vaping? Marketed as the healthier alternative, it comes in bright colours, fruity flavours, and without the sharp reminder of what our lungs might look like in a few years’ time. We are keen to stay at the cutting edge of the latest, modern, trendy technology, and vaping is the latest in a long line of products that enables us to do so. We are products of our environment, and it shows up in individual attitudes to drinking and smoking. In friend groups that dislike the smell of cigarettes, or in homes with parents who don’t approve of drinking, it is far easier to avoid them. Yet in social circles that explain away vaping as ‘just flavoured air’, or who joke about people not being able to handle their drink, it is far harder to resist and to be the only person without a drink or a vape in their hand. This subtler peer pressure is far removed from the extreme examples used in schools, of friends actively encouraging or forcing huge consumption.

Social media gives us an insight into other people’s lives, anywhere in the world. As young people, we often hold celebrities and influencers up as role models, seeking to emulate them. They often endorse a variety of products, or appear in adverts, but they also show an element of their personal lives. Vaping, smoking and drinking are rarely outright promoted, but they often feature subtly in posts – a bottle in the corner of a photo of a concert, or a cigarette in a hand in a group photo. The aim of the influencers is not necessarily to promote these things, but nevertheless, they add to the sense of rebellious glamour that already surrounds alcohol and nicotine.

Additionally, we often don’t consider the aim of the company behind the app. Naturally, their goal is to keep users on their app for as long as possible, so they can show them as many adverts as possible, and make as much money from them as possible. This results in algorithms targeted as specifically as possible to your interests, something that you’ll notice here and there if you engage with a post on a specific topic – suddenly it’s all over your feed. These algorithms try and suggest content related to things you’ve enjoyed before, which sounds great until it starts to suggest gambling pages because you’ve taken an interest in a sport.

Though smoking, drinking, vaping and fast food have become ingrained in our societies, we can still change the way that we interact with them. The previously mentioned change in legislation for cigarette packaging in 2016 has been effective at reducing the number of people taking up smoking, and we would support the same idea being extended to alcohol and vapes. Removing the colourful packaging, which seems designed to appeal specifically to young people, and including clear, concise labelling of the health risks associated with consumption will enable us to make more level-headed, informed decisions about our purchasing. Many people still smoke cigarettes despite their packaging, so this would instead be primarily aimed at reducing the number of new consumers trying vapes for the first time. There is the challenge of deciding precisely which components to regulate; coils, salts, liquids, pods and the vaping device itself are all sold separately, and unlike cigarettes, the packaging is often thrown away shortly after purchase. A common suggestion from those that we spoke to was legislation restricting the design of the vaping device itself; moving away from the bright colours and flashing lights that make many of the currently available vapes look closer to children’s toys than anything else. The general opinion was that the individual components are all used for the sole purpose of vaping and should therefore be subject to regulation. By treating vapes in the same way as tobacco, and storing them in closed cupboards out of sight, only available on request, we can reduce their appeal to younger generations who see them in the shop windows on their way home from school, and reduce the frequency of underage consumers shoplifting them.

Alcohol and tobacco are already subject to increased taxation, and treating vapes as an extension of tobacco products seems a simple next step. However, this obviously affects the least wealthy among us the most, who have the least choice in their consumption, and further drives wealth inequalities. Higher prices, or at the extreme an outright ban, are likely to drive people to alternative sources, perhaps through illicit means, or to seek out substitutes in the same way that vaping has replaced smoking.

While children’s television advertising is restricted, many children are moving away from television and on to social media or streaming services, where they often see the same content as an adult sees. Phones and social media should be carefully considered in the creation of new policies, particularly for their role in marketing the other products discussed here. Content produced by individual influencers is hard to regulate and monitor due to the sheer volume, but there already exists a variety of systems to monitor company-sponsored advertising. By creating an effective age-verification scheme on social media, we can restrict the types of advertisements that are shown to young people, in a similar way to the television watershed. Likewise, restricting the types of advertising shown in conjunction with sports programmes, which are often watched by children, to a more family-friendly equivalent that doesn’t feature fast food and gambling brands would be beneficial.

Many of us, young people or otherwise, are unaware of the risks associated with social drinking, or of consumption in small volumes. However, the existing resources available to schools to educate us on this are often financially linked to the alcohol industry; such as SMASHED, which is funded by DIAGEO (‘a global leader in beverage alcohol’)^2^ as part of their initiatives to ‘promote positive drinking’.^3^ These often focus on individual rather than corporate responsibility, granting the industry the opportunity to present itself as part of the solution. As such, independent public health campaigns designed to demonstrate the risks and damages associated with varying levels of consumption, so that the public can make informed decisions about their health, would fill a much-needed gap. Improved education on smoking, vaping and gambling is also important but, as with alcohol, it can be challenging to create an engaging discussion around the topic, as so many young people are mentally switched off by even the mention of the topic. This could be addressed by using examples of real people, showing young people the damage that their habits can cause, hopefully alarming them into making a change in their habits. Likewise, teaching critical thinking in schools to give us the skills to be able to see through marketing ploys applies to all areas of life, and sets us up for success in the future.

## CRediT authorship contribution statement

**Joseph Dickenson:** Writing – review & editing, Writing – original draft, Conceptualization. **Sean Devlin:** Writing – review & editing, Writing – original draft, Conceptualization.

## Declaration of competing interest

The authors declare that they have no known competing financial interests or personal relationships that could have appeared to influence the work reported in this paper.
